# Rethinking the Structure of Global Surgical Education: Making the Case for ICOSET

**DOI:** 10.1002/wjs.70465

**Published:** 2026-06-21

**Authors:** Amit R. T. Joshi, Adrian Anthony, Dara O'Keeffe, April Roslani, David O'Regan

**Affiliations:** ^1^ Cooper Medical School of Rowan University Cooper University Healthcare Camden New Jersey USA; ^2^ The Queen Elizabeth Hospital Adelaide South Australia Australia; ^3^ Royal College of Surgeons in Ireland Dublin Ireland; ^4^ Universiti Malaya Kuala Lumpur Kuala Lumpur Malaysia

Surgical education has entered an era in which its challenges are no longer local [1]. Workforce shortages, competency‐based training paradigms, regulatory complexity, generational factors and rapid technological change are shared across countries and health systems [2]. Yet the structures that govern surgical education remain largely national or institution‐specific. This mismatch between global challenges and local organizational frameworks is increasingly difficult to ignore [3, 4, 5].

Traditional organizational models have each contributed meaningfully to surgical education. Professional colleges provide institutional continuity, legitimacy, and infrastructure. Specialty societies have demonstrated agility, while certifying bodies ensure standards and accountability. However, none of these models was designed to function as a truly international educational platform [6].

A different structural approach is therefore required. Federated, society‐based organizations offer a compelling alternative. This model enables representation across diverse regions while preserving local autonomy and facilitating bidirectional knowledge exchange. A practical example of this federated structure is seen in the participating society model of the International Society of Surgery (ISS). Within this framework, national, regional, and specialty societies engage as integrated partners contributing to a shared enterprise. Participation extends beyond attendance at congresses to include co‐development of programming, workshops, and educational strategy. This structure allows societies to retain autonomy while benefiting from global collaboration and visibility.

The International Collaborative of Surgical Education and Training (ICOSET) was conceived in the mid‐2000s by an international group of surgical educators representing major colleges across multiple regions. ICOSET began as a response to shared challenges in training, including competency‐based education, evolving regulatory expectations, and increasing complexity in surgical practice. Initially structured as a biennial conference, it convened educators, trainees, and institutional leaders in a collaborative environment focused on educational exchange.

Over successive meetings across Europe, North America, and Australasia, ICOSET developed a track record of addressing simulation, patient safety, professionalism, and educational design while generating consensus statements on surgical training. This period of intellectual exchange established both credibility and a networked community, creating the foundation for organizational transition.

The transition of ICOSET in 2025 from a conference‐based initiative to a registered nonprofit organization marked a critical inflection point. Governance structures were formalized through a Board of Trustees and designated officer roles to ensure accountability and continuity. ICOSET now functions as a collaborative international network linking surgical societies, colleges, certifying boards, training programs, educators, trainees, and students across diverse health systems [7]. Its emphasis is not certification, but the advancement of teaching, simulation, assessment, and faculty development through practical educational collaboration. The organization aims to support co‐development of courses, workshops, webinars, and digital educational resources that can be adapted to local contexts while maintaining shared educational principles. In doing so, ICOSET seeks to connect local training efforts to a broader international educational community, making high‐quality surgical education more accessible, relevant, and responsive to patient needs worldwide.

A defining feature of ICOSET is its recognition of the social contract of surgery. Educational systems must align with the needs of the communities they serve. Training must be adaptable to local contexts while maintaining standards that ensure patient safety and professional competence.

The tension between standardization and adaptation is unavoidable. Federated structures are uniquely suited to managing this tension by preserving flexibility in implementation while maintaining shared principles. Financial considerations are equally important. Many international organizations rely heavily on annual congresses as their primary revenue source. ICOSET's early financial model, characterized by low fixed costs and self‐funding, reflects both constraint and opportunity. The future trajectory of ICOSET will depend on execution. The organization must translate intent into operational value through longitudinal programming, digital platforms, and meaningful engagement across stakeholders.

No model is without limitations. Federated systems depend on the engagement and capacity of member societies. However, these challenges are inherent to the complexity of global surgery. ICOSET should therefore be understood not as a replacement for existing institutions but as a complementary structure addressing the gap between local training systems and global educational needs.

The next phase of global surgical education will be defined by structure as much as content. Federated models provide a viable framework, but their success depends on active participation. The central question remains: can a surgeon anywhere in the world access the education needed to provide optimal care when and where it is required? ICOSET represents a meaningful step in that direction.

## Author Contributions

**Amit R. T. Joshi:** conceptualization, methodology, formal analysis, writing – original draft, writing – review and editing. **Adrian Anthony:** writing – original draft, writing – review and editing. **Dara O’Keeffe:** writing – review and editing. **April Roslani:** writing – review and editing. **David O’Regan:** writing – original draft, conceptualization, writing – review and editing, investigation.

## Funding

The authors have nothing to report.

## Data Availability

Data sharing not applicable to this article as no datasets were generated or analyzed during the current study.
